# The Etiological Profile of Neonatal Thrombocytopenia in Neonates in Neonatal Intensive Care Unit: A Cross-Sectional Study

**DOI:** 10.7759/cureus.48422

**Published:** 2023-11-07

**Authors:** Aboli Dahat, Girish Nanoti, Manish Chokhandre, Heena Bhandekar

**Affiliations:** 1 Department of Pediatrics, Narendra Kumar Prasadrao (NKP) Salve Institute of Medical Sciences and Research Centre and Lata Mangeshkar Hospital, Nagpur, IND; 2 Department of Pediatrics, Datta Meghe Medical College and Shalinitai Meghe Hospital and Research Centre, Nagpur, IND

**Keywords:** necrotizing enterocolitis, respiratory distress syndrome, disseminated intravascular coagulation, prematurity, sepsis, thrombocytopenia, nicu

## Abstract

Background

Neonatal thrombocytopenia is one of the most common clinical entities encountered in the neonatal intensive care unit (NICU); if not identified early, it can lead to significant morbidity and mortality. The aim of this study was to find out the etiological profile of neonatal thrombocytopenia in the NICU and to study the association between the etiology and onset of thrombocytopenia.

Methods

It was a single-center, cross-sectional, descriptive study of neonates having thrombocytopenia. The study was carried out in the NICU of the department of pediatrics in a tertiary care center over a period of one year. The study population included neonates admitted to the NICU having thrombocytopenia (platelet count: <150×10^9^/L). The demographic data such as name, sex, gestational age, age at the onset of thrombocytopenia, and birth weight was recorded. Data was collected based on laboratory investigations.

Results

Early-onset thrombocytopenia was present in 34% of neonates, and late onset was seen in (66%). A statistically significant result was found in disseminated intravascular coagulation (DIC); the p value was 0.02. The majority of neonates had late-onset sepsis (LOS) (57%). In both early-onset sepsis (EOS) and LOS, 36.84% each, the majority of neonates had moderate thrombocytopenia. Statistically significant results were found in respiratory distress syndrome (RDS) and necrotizing enterocolitis (NEC); the p value was 0.004 and 0.03, respectively.

Conclusion

Thrombocytopenia is a universal finding in neonates in the NICU, and it is an important prognostic marker of various disease conditions in neonates. Thus, the timely recognition and management of thrombocytopenia is essential to reduce neonatal morbidity and mortality.

## Introduction

Thrombocytopenia is one of the frequent and universal hematopoietic entities found in neonates in the neonatal intensive care unit (NICU) [1]. Neonatal thrombocytopenia is characterized by a platelet count of <150×10^9^/L in any neonate of viable gestational age. The incidence of thrombocytopenia in newborns is 1%-5% at birth, while severe thrombocytopenia exists in 0.1%-0.5% of neonates. The incidence of thrombocytopenia is inversely proportional to the gestational age and birth weight of a newborn. Most of the neonates in NICU manifest mild (100-150×10^9^/L) to moderate (50-99×10^9^/L) thrombocytopenia, while in about 20% of neonates, severe thrombocytopenia (<50×10^9^/L) is seen. Neonates are more predisposed to develop thrombocytopenia in response to illness, which may be because of the limited ability of the neonatal megakaryopoietic axis to increase the production of platelets in response to platelet consumption [2].

The pathophysiology of neonatal thrombocytopenia is similar to that of adults, which consists of decreased platelet production, increased platelet consumption, hypersplenism, or a combination of all of these mechanisms. The etiological factors responsible for neonatal thrombocytopenia are prematurity, birth asphyxia, intrauterine growth retardation (IUGR), low birth weight (LBW), hyperbilirubinemia, meconium aspiration syndrome (MAS), respiratory distress syndrome (RDS), and sepsis. Maternal disseminated intravascular coagulation (DIC) and pregnancy-induced hypertension (PIH) have also contributed to the etiology of neonatal thrombocytopenia. Severe thrombocytopenia is largely manifested in preterm babies with a gestational age of less than 36 weeks, extremely low birth weight babies (ELBW, <1000 g), or critically ill neonates in the NICU [1].

The causative factors responsible for early-onset thrombocytopenia (first 72 hours of life) are different from those of late-onset thrombocytopenia (after 72 hours of life) [3]. Most of the episodes of neonatal thrombocytopenia are relatively self-resolving and mild, and they last for a shorter duration. However, sometimes, it may cause morbidity and mortality because of serious complications such as intraventricular hemorrhage (IVH) [4]. The incidence of IVH was as high as 60% in neonates with severe thrombocytopenia, and mortality was directly proportional to the severity of thrombocytopenia. Since the etiology determines the clinical course and outcome, early identification and appropriate diagnostic evaluation are essential for the improved survival of the neonates [4].

## Materials and methods

It was a single-center, cross-sectional, descriptive, and observational study carried out on 100 neonates in the NICU of the department of pediatrics in a tertiary care center. The study population was neonates admitted to the NICU having thrombocytopenia (platelet count: <150×109/L), while neonates having chromosomal or genetic disorders and neonates born outside the hospital having thrombocytopenia and who received platelet transfusion in other NICU were excluded from the study. The sample size for this study was calculated with reference to statistical data [2] using the following formula: n=(Z²*p*(1-p))/E².

After obtaining the institutional ethics committee's (IEC) approval and the parents' or guardians' written informed consent (IEC number: 7/2021), neonates were recruited for the study. The study was conducted from January 2021 to December 2021, and the duration of the study was one year.

The demographic data such as name, sex, gestational age, age at the onset of thrombocytopenia, and birth weight was recorded. Information was procured from parents/guardians of the enrolled neonates, and the details of demographic and clinical parameters were noted. A structured interviewer-administered questionnaire was used as the data-collecting tool; it had been pretested and modified before being used in the study. Data was collected based on laboratory investigations. The sample was collected using the convenience sampling technique. The blood specimens were collected from each neonate before the administration of antibiotics. Blood samples were obtained for the sepsis workup, which included total leukocyte count (TLC), absolute neutrophil count (ANC), immature neutrophils to total neutrophil count ratio (I/T ratio), platelet count, blood culture and antibiotic sensitivity, and C-reactive protein (CRP) estimation. One milliliter of blood was drawn and then put up in the autoanalyzer (Siemens ADVIA 120 autoanalyzer, Berlin, Germany) for the analysis of the blood for complete blood count, differential leukocyte count, and platelet count. The machine works on the flow cytometry principle. A scattergram was used to reduce the platelet bias.

Neonatal thrombocytopenia is characterized by a neonate with a platelet count of less than 1.5 lakh/cumm, which is further classified into the following [1]: a) mild thrombocytopenia, 1-1.5 lakh/cumm; b) moderate thrombocytopenia, 50000-99000/cumm; and c) severe thrombocytopenia, <50000/cumm.

Neonatal thrombocytopenia can also be classified based on the timing of presentation as early and late, which is used in the diagnostic evaluation [1]: a) Early thrombocytopenia is defined as thrombocytopenia that occurs within the first 72 hours of life, and b) late thrombocytopenia is defined as thrombocytopenia that occurs 72 hours after delivery.

Structured data-collecting forms were used to gather the data. Every observation and discovery were coded, recorded into a Microsoft (MS) Excel (Microsoft® Corp., Redmond, WA) master spreadsheet, and then analyzed with the Epi Info software (Centers for Disease Control and Prevention, Atlanta, GA). Analysis was conducted using the Statistical Package for Social Sciences (SPSS) version 25 (IBM SPSS Statistics, Armonk, NY) and Epi Info version 7.3. The chi-square test and Fischer's exact test were used to see the association between independent variables.

## Results

**Figure 1:**
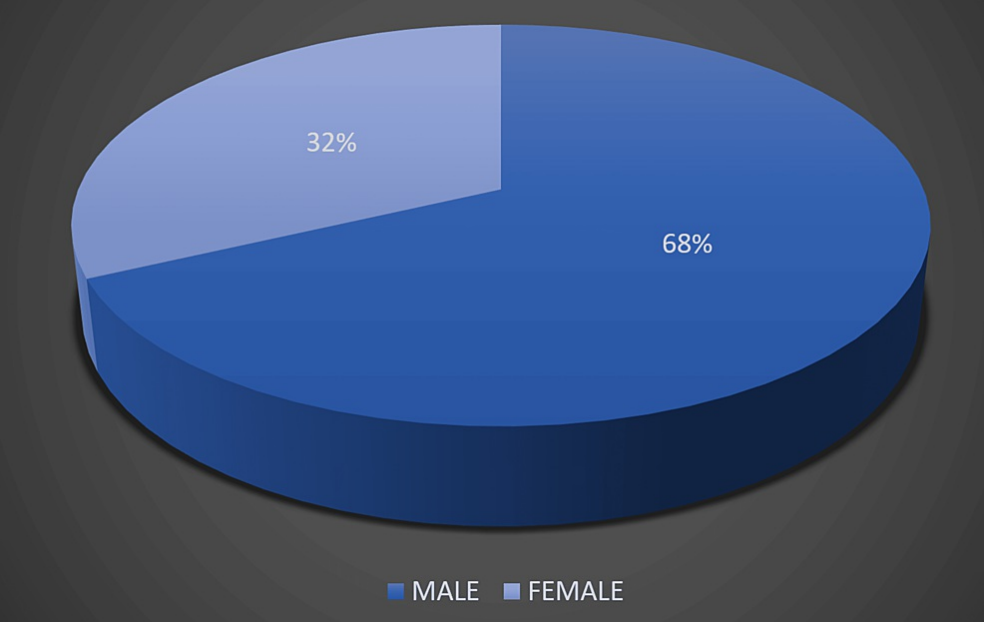
Distribution of study subjects according to gender

Figure 1 shows the distribution of study subjects according to gender. In our study, males outweighed females in numbers. Out of 100 neonates, 75% (75) constituted the preterm neonates (<37 weeks), and term neonates (37-42 weeks) constituted the remaining.

**Figure 2:**
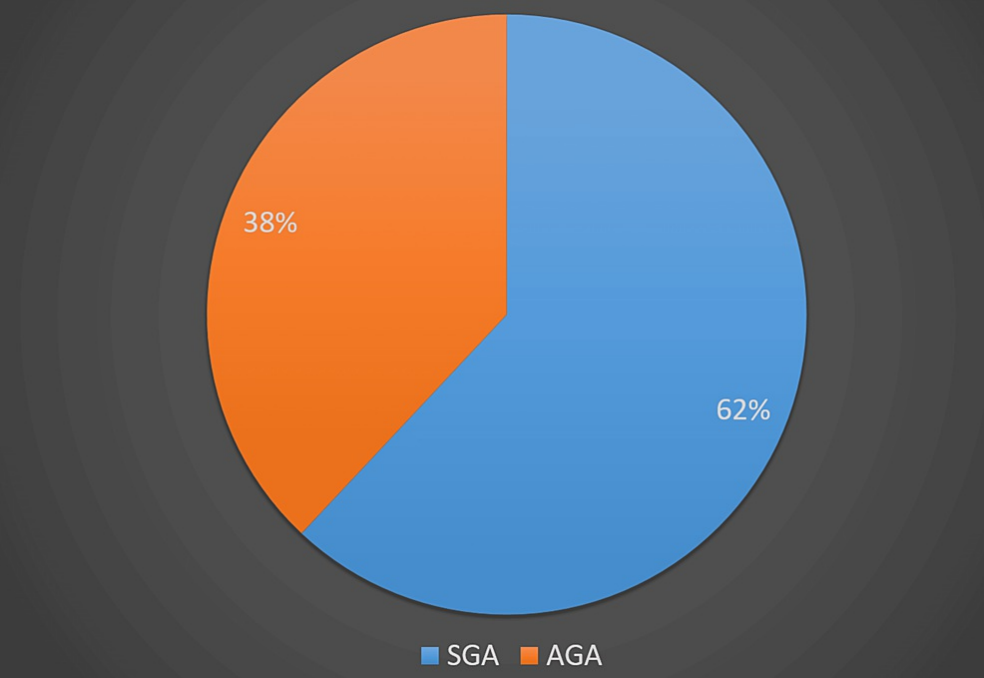
Distribution of neonates according to whether appropriate for gestational age (AGA) SGA: small for gestational age

Figure 2 shows the distribution of study subjects according to whether appropriate for gestational age (AGA). Sixty-two (62%) subjects were appropriate for gestational age (AGA), and the rest (38, 38%) were small for gestational age (SGA), and there was no large for gestational age (LGA) baby in our group.

Table 1 shows the distribution of the neonates according to the severity of thrombocytopenia. The majority of neonates had moderate thrombocytopenia (37, 37%), while mild and severe thrombocytopenia was seen in 32 (32%) and 31 (31%) neonates, respectively.

**Table 1:** Distribution of neonates according to the type of thrombocytopenia

Thrombocytopenia (lakhs/cumm)	Number	Percentage
Mild	32	32
Moderate	37	37
Severe	31	31

Table 2 shows the etiology of thrombocytopenia. The most common causative factor of thrombocytopenia was sepsis (95%), followed by prematurity (75%), RDS (21%), DIC (20%), necrotizing enterocolitis (NEC) (7%), and perinatal asphyxia (7%). Early-onset sepsis (EOS) was seen 33 (34.74%) and late-onset sepsis (LOS) were seen in 62 (65.26%) neonates. Culture-positive EOS was seen in 66.66%, and culture-positive LOS was seen in 93.54%.

**Table 2:** Distribution of neonates according to etiology EOS, early-onset sepsis; LOS, late-onset sepsis; RDS, respiratory distress syndrome; NEC, necrotizing enterocolitis; DIC, disseminated intravascular coagulopathy; ITP, idiopathic thrombocytopenic purpura; NAIT, neonatal alloimmune thrombocytopenia

Etiology	Number	Percentage
Sepsis	95	95
EOS	33	34.74
LOS	62	65.26
RDS	21	21
NEC	7	7
DIC	20	20
Prematurity	75	75
Neonatal hyperbilirubinemia	1	1
Metabolic disorder	0	0
Maternal ITP (autoimmune)	0	0
NAIT	0	0

Table 3 shows the association between the etiology and onset of thrombocytopenia. In neonatal sepsis, (95, 95%), early-onset thrombocytopenia was present in 33 neonates (34.74%), and late onset was present in 62 (65.26%) neonates. Late-onset thrombocytopenia (47, 62.67%) was more predominant in preterm neonates as compared to early-onset thrombocytopenia, which was seen in 28 (37.33%) neonates. Using Fisher's exact test, a statistically significant result was obtained in DIC (p value of 0.02), in which out of 20 neonates, early-onset thrombocytopenia was present in 11 (55%) neonates and nine (44%) neonates presented with late-onset thrombocytopenia.

**Table 3:** Association between the etiology and onset of thrombocytopenia in neonates *Statistically significant result was obtained in DIC (p value of 0.02) SGA, small for gestational age; RDS, respiratory distress syndrome; NEC, necrotizing enterocolitis; DIC, disseminated intravascular coagulation

Etiology	Onset of thrombocytopenia		p value
	Early onset (N=34), number (%)	Late onset (N=66), number (%)	
SGA (n=38)	17 (44.74)	21 (55.26)	0.07
Perinatal asphyxia (n=07)	1 (14.25)	6 (85.71)	0.41
RDS (n=21)	7 (33.33)	14 (66.67)	0.94
Neonatal sepsis (n=95)	33 (34.74)	62 (65.26)	0.49
NEC (n=07)	3 (42.86)	4 (57.14)	0.60
DIC (n=20)	11 (55)	9 (44)	0.02*
Preterm (n=75)	28 (37.33)	47 (62.67)	0.22

Table 4 shows the association between neonatal risk factors and the type of thrombocytopenia. Maximum neonates were having neonatal sepsis (95%), out of which 38 neonates had early-onset sepsis and 57 had late-onset sepsis. In both early- and late-onset sepsis, most of the neonates had moderate thrombocytopenia, 14 (36.84%) and 21 (36.84%), respectively. Using Fisher's exact test, a statistically significant result was obtained in RDS and NEC. The p value was 0.004 and 0.03, respectively. In RDS, out of 21 neonates, the majority had moderate thrombocytopenia (13, 61.9%), while in NEC, out of seven neonates, severe thrombocytopenia was seen in five (78.43%) neonates, and the remaining two (28.57%) had moderate thrombocytopenia.

**Table 4:** Association between neonatal risk factors and the type of thrombocytopenia *Statistically significant result was obtained in RDS and NEC. The p value was 0.004 and 0.03, respectively SGA, small for gestational age; EOS, early-onset sepsis; RDS, respiratory distress syndrome; LOS, late-onset sepsis; NEC, necrotizing enterocolitis; DIC, disseminated intravascular coagulation

Risk factors	Thrombocytopenia			
	Mild, N (%)	Moderate, N (%)	Severe, N (%)	p value
SGA (n=38)	10 (26.32)	16 (42.11)	12 (31.58)	0.58
Perinatal asphyxia (n=07)	3 (42.85)	1 (14.28)	3 (42.85)	0.43
EOS (n=38)	12 (31.58)	14 (36.84)	12 (31.58)	0.99
RDS (n=21)	1 (4.76)	13 (61.90)	7 (33.34)	0.004*
LOS (n=57)	17 (29.82)	21 (36.84)	19 (33.33)	0.80
NEC (n=07)	0 (0)	2 (28.57)	5 (71.43)	0.03*
DIC (n=20)	6 (30)	7 (35)	7 (35)	0.91
Preterm (n=75)	26 (34.67)	30 (40)	19 (25.33)	0.10

Table 5 shows the distribution of neonates according to the onset of sepsis. Out of 100, 38 (38%) neonates had EOS. LOS was seen in 57 (57%) neonates, while in five (5%) neonates, there was no sepsis.

**Table 5:** Distribution of neonates according to the onset of sepsis EOS, early-onset sepsis; LOS, late-onset sepsis

Onset of sepsis	Number	Percentage
EOS	38	38
LOS	57	57
No sepsis	5	5
Total	100	100

## Discussion

Thrombocytopenia is one of the most prevalent and universal hematopoietic conditions that can be seen in newborns in the NICU [1]. In fetal circulation, platelets start appearing in circulation by around the fifth week of gestation and reach a normal adult level of 150-450×10^9^/L by the second trimester of gestation, and then, the same level persists throughout life. Though it has been recognized that the presence of severe thrombocytopenia (platelets of <50×10^9^/L) in neonates requires special clinical recognition, the relationship between the severity of thrombocytopenia and the likelihood of bleeding is relatively lacking [5].

In our study, we found that thrombocytopenia was predominantly present in preterm babies (75, 75%) as compared to term babies (25%). Similar results were seen by Madhavi et al. [4] and Sharma and Thapar [6]; in their studies, 63.3% and 58.2% of preterm neonates developed thrombocytopenia, respectively. Moderated thrombocytopenia predominated in preterm babies in our study, while in a study done by Meena et al. [7], they found that severe thrombocytopenia was predominantly seen in preterm babies whereas term babies had moderate thrombocytopenia. Thrombocytopenia is rarely severe and is independently associated with prematurity and lesser gestational age at birth [8]. In our study, 62 (62%) neonates were AGA, and the rest (38, 38%) were SGA. Moderate thrombocytopenia was predominantly seen in SGA neonates. Identical findings were obtained by Fustolo-Gunnink et al., who showed that 53% of SGA and 20% of AGA neonates had thrombocytopenia and the severity of thrombocytopenia was higher in the SGA group. They found that early-onset thrombocytopenia predominated SGA neonates (50%) and its frequency was 2.7 times more as compared to AGA neonates. Thrombocytopenia is frequently seen in SGA neonates, and it usually occurs due to the phenomena of impaired megakaryopoiesis leading to decreased platelet production [8].

In our study, males outweighed females in numbers. Thrombocytopenia was more seen in males (68, 68%) as compared to females (32, 32%). Similar results were obtained by Tirupathi et al. [9] and Rathi [5]; in their study, 56% and 59% were males, and 44% and 41% were females, respectively. While opposite results were seen by Addil et al. [10], they found that thrombocytopenia was present in 68.9% of neonates and was predominantly seen in females. It has been observed that in most of the studies of neonatal thrombocytopenia, males have predominance over females; however, the cause for such distribution is not yet clear. The majority of neonates in our study had moderate thrombocytopenia (37, 37%); however, mild thrombocytopenia was seen in 32 (32%), and the rest of the neonates (31, 31%) had severe thrombocytopenia. In a study done by Meena et al. [7], most of the neonates had mild thrombocytopenia (46%), followed by moderate (35%) and then severe thrombocytopenia (19%). Similar results were found by Sanii et al. [11]; in their study, mild, moderate, and severe thrombocytopenia was present in 43.5%, 25.8%, and 24.1% of neonates, respectively. It shows that severe thrombocytopenia is an infrequent variety. Most of the neonates usually present with mild to moderate variety, while severe thrombocytopenia is seen in approximately 20% of neonates [2].

The etiological profile of our study showed neonatal sepsis (95, 95%) as the most common cause of thrombocytopenia in neonates; subsequently, prematurity (75, 75%), SGA (38, 38%), and RDS (21, 21%) were the major causes. Likewise, Gupta et al. [3], Sanii et al. [11], and Madhavi et al. [4] also found sepsis to be the most frequent cause of neonatal thrombocytopenia, which manifested in 42%, 24.1%, and 74.5% of neonates, respectively. While as per the study done by Jeremiah and Oburu [12], the most common etiologies were birth asphyxia (33.3%) and jaundice (19.7%). Thus, sepsis, prematurity, birth asphyxia, and lower gestational age are common and independent variables that could lead to neonatal thrombocytopenia.

In this study, early-onset thrombocytopenia was present in 34 (34%) neonates, while late onset was present in 66 (66%). Other than DIC, all other risk factors had late-onset thrombocytopenia, while in neonatal sepsis complicated with DIC, it was early-onset thrombocytopenia, which was statistically significant (p value of 0.02). Similar results were found by Nandyal et al. [13]; in their study, early-onset thrombocytopenia was present in 43.4% of neonates, while the remaining 56.5% of neonates presented with late-onset thrombocytopenia. Both sepsis and birth asphyxia were associated with late-onset thrombocytopenia. Similarly, in a study done by Meena et al. [7], early-onset thrombocytopenia was seen in 51%, while the remaining neonates (49%) had late-onset thrombocytopenia. In their study, birth asphyxia and sepsis were both strongly related to early- and late-onset thrombocytopenia, respectively.

We found that the incidence of LOS (57, 57%) was more as compared to EOS (38, 38%), while there was no evidence of sepsis in five (5%) neonates. Similar results were obtained by Lim et al. [14], with LOS compromising the majority (93.7%). While in a study done by Bagale and Bhandari [15], the majority (27%) of neonates presented with EOS, while LOS was seen in only 2.7%. In our study, moderate thrombocytopenia predominated in both early- and late-onset sepsis, 14 (36.84%) and 21 (36.84%), respectively. However, severe thrombocytopenia was more prevailing in late-onset sepsis as compared to early-onset sepsis. In a study done by Bagale and Bhandari [15], 33.3% of neonates who manifested with severe thrombocytopenia were found to have sepsis, while in mild thrombocytopenic neonates, sepsis accounted for 27.9%, while asphyxia accounted for 37.2%.

Though the common causative factors for early-onset thrombocytopenia are chronic fetal hypoxia, NEC and sepsis have been found to be the important risk factors for late-onset thrombocytopenia. However, no etiology was identified in a significant proportion of thrombocytopenia in ELBW neonates [16]. Mortality increases by four times in neonatal sepsis associated with thrombocytopenia, and it will further increase by six times in the case of Gram-negative sepsis [17]. As one in every four neonates manifests thrombocytopenia at one point during their NICU stay, it becomes a constant challenge for pediatricians and neonatologists for the early assessment, evaluation, and timely intervention of the condition [11].

Failure to include the clinical profile and detailed maternal history in the study was the limitation of the study. The referral of neonates from outside of the study setting who already presented with sepsis could have led to selection bias.

It was a single-center, small-scale study where the sample size was few. A multicentric study with a large sample size needs to be done for a detailed understanding of the etiology of thrombocytopenia considering both maternal and neonatal factors that are responsible for thrombocytopenia, which will ultimately lead to a decrease in morbidity and mortality of neonates.

## Conclusions

Neonatal thrombocytopenia is a frequent and universal clinical finding encountered in neonates admitted to the NICU, and it serves as a key prognostic indicator of various disease conditions in newborns admitted to the NICU. Thus, the early identification, evaluation, and timely intervention of thrombocytopenia are essential to reduce morbidity and mortality. Sepsis and prematurity were discovered to be separate independent causal factors for poor prognosis in newborns admitted to the NICU. As thrombocytopenia is relatively common in newborns, it is crucial to check the platelet count, pattern of onset, and degree and severity of thrombocytopenia in every newborn admitted to the NICU. This will aid the neonatologist in making a diagnosis, planning interventions, and starting treatment early and promptly.

## References

[1] Roberts I, Murray NA (2003). Neonatal thrombocytopenia: causes and management. Arch Dis Child Fetal Neonatal Ed.

[2] Sola MC, Del Vecchio A, Rimsza LM (2000). Evaluation and treatment of thrombocytopenia in the neonatal intensive care unit. Clin Perinatol.

[3] Gupta A, Mathai SS, Kanitkar M (2011). Incidence of thrombocytopenia in the neonatal intensive care unit. Med J Armed Forces India.

[4] Madhavi D, Subuhi S, Zubair M (2020). Outcome of neonatal thrombocytopenia in tertiary care NICU. J Pediatr Neonatal Care.

[5] Rathi P (2021). Clinical profile and outcome of neonatal thrombocytopenia in a tertiary care hospital. Medpulse Int J Pediatr.

[6] Sharma A, Thapar K (2015). A prospective observational study of thrombocytopenia in high risk neonates in a tertiary care teaching hospital. Sri Lanka J Child Health.

[7] Meena SL, Singh K, Jain S, Jain A, Karnawat BS (2019). Clinical profile and outcome of neonatal thrombocytopenia in a tertiary care hospital. Int J Contemp Pediatr.

[8] Fustolo-Gunnink SF, Vlug RD, Smits-Wintjens VE, Heckman EJ, Te Pas AB, Fijnvandraat K, Lopriore E (2016). Early-onset thrombocytopenia in small-for-gestational-age neonates: a retrospective cohort study. PLoS One.

[9] Tirupathi K, Swarnkar K, Vagha J (2017). Study of risk factors of neonatal thrombocytopenia. Int J Contemp Pediatr.

[10] Addil F, Rehman A, Najeeb SM, Imtiaz H, Khan SE (2021). Neonatal sepsis: the frequency of thrombocytopenia. Sys Rev Pharm.

[11] Sanii S, Khalessi N, Khosravi N, Mehrjerdi FZ (2013). The prevalence and risk factors for neonatal thrombocytopenia among newborns admitted to intensive care unit of aliasghar children's hospital. Iran J Blood Cancer.

[12] Jeremiah Z, Oburu J (2010). Pattern and prevalence of neonatal thrombocytopenia in Port Harcourt, Nigeria. Pathol Lab Med Int.

[13] Nandyal SS, Shashikala P, Sahgal V (2016). Study of thrombocytopenia in neonatal intensive care unit. Indian J Pathol Oncol.

[14] Lim WH, Lien R, Huang YC (2012). Prevalence and pathogen distribution of neonatal sepsis among very-low-birth-weight infants. Pediatr Neonatol.

[15] Bagale BB, Bhandari A (2018). Neonatal thrombocytopenia: its associated risk factors and outcome in NICU in a tertiary hospital in Nepal. J Coll Med Sci Nepal.

[16] Uhrynowska M, Maslanka K, Zupanska B (1997). Neonatal thrombocytopenia: incidence, serological and clinical observations. Am J Perinatol.

[17] Ree IM, Fustolo-Gunnink SF, Bekker V, Fijnvandraat KJ, Steggerda SJ, Lopriore E (2017). Thrombocytopenia in neonatal sepsis: Incidence, severity and risk factors. PLoS One.

